# Lymphatic Endothelial Cell Defects in Congenital Cardiac Patients With Postoperative Chylothorax

**DOI:** 10.1097/jova.0000000000000016

**Published:** 2021-09

**Authors:** Aqsa Shakoor, June K. Wu, Ajit Muley, Christopher Kitajewski, Joseph D. McCarron, Noa Shapiro-Franklin, Rozelle Corda, Sophia Chrisomalis-Dring, Paul J. Chai, Carrie J. Shawber

**Affiliations:** a Division of Pediatric Surgery, Department of Surgery, Columbia University Irving Medical Center Vagelos College of Physicians & Surgeons and New York Presbyterian Hospital/Morgan Stanley Children’s Hospital, New York, New York;; b Division of Plastic Surgery, Department of Surgery, Columbia University Irving Medical Center Vagelos College of Physicians & Surgeons and New York Presbyterian Hospital/Morgan Stanley Children’s Hospital, New York, New York;; c Division of Reproductive Sciences, Department of Ob/Gyn, Columbia University Irving Medical Center Vagelos College of Physicians & Surgeons and New York Presbyterian Hospital/Morgan Stanley Children’s Hospital, New York, New York;; d Division of Cardiothoracic Surgery, Department of Surgery, Columbia University Irving Medical Center Vagelos College of Physicians & Surgeons and New York Presbyterian Hospital/Morgan Stanley Children’s Hospital, New York, New York;; e Division of Pediatric Cardiology, Department of Pediatrics, Columbia University Medical Center Vagelos College of Physicians & Surgeons, and New York Presbyterian/Morgan Stanley Children’s Hospital, New York, New York

**Keywords:** Chylothorax, Lymphatic endothelial cells, Congenital cardiac anomalies

## Abstract

**Objectives:**

Chylothorax following cardiac surgery for congenital cardiac anomalies is a complication associated with severe morbidities and mortality. We hypothesize that there are intrinsic defects in the lymphatics of congenital cardiac patients.

**Methods:**

Postsurgical chylothorax lymphatic endothelial cells (pcLECs) (n = 10) were isolated from the chylous fluid from congenital cardiac defect patients, and characterized by fluorescent-activated cell sorting, immunofluorescent staining, and quantitative RT-PCR. Results were compared to normal human dermal lymphatic endothelial cells (HdLECs). pcLECs (n = 3) and HdLECs were xenografted into immunocompromised mice. Implants and postoperative chylothorax patient’s pulmonary tissues were characterized by immunostaining for lymphatic endothelial proteins.

**Results:**

pcLECs expressed endothelial markers VECADHERIN, CD31, VEGFR2, lymphatic endothelial markers PROX1, podoplanin, VEGFR3, and progenitor endothelial markers CD90 and CD146. However, pcLECs had key differences relative to HdLECs, including altered expression and mislocalization of junctional proteins (VECADHERIN and CD31), and essential endothelial proteins, VEGFR2, VEGFR3, and PROX1. When xenografted in mice, pcLECs formed dilated lymphatic channels with poor cell–cell association. Similar to congenital lymphatic anomalies, the pulmonary lymphatics were dilated in a patient who developed postoperative chylothorax after cardiac surgery.

**Conclusions:**

Recent studies have shown that some postoperative chylothoraces in congenital cardiac anomalies are associated with anatomical lymphatic defects. We found that pcLECs have defects in expression and localization of proteins necessary to maintain lymphatic specification and function. This pcLEC phenotype is similar to that observed in lymphatic endothelial cells from congenital lymphatic anomalies. Co-existence of lymphatic anomalies should be considered as a feature of congenital cardiac anomalies.

## Introduction

Secondary chylothorax, defined as postoperative pleural effusion after pediatric cardiothoracic surgery to correct congenital, structural cardiac defects, has an incidence of 0.85%–6.6%.^1–3^ The characteristics of postoperative chylous effusion fluid include lymphocytic predominance and high triglyceride count.^4,5^ Secondary chylothoraces result in significant morbidities, including prolonged ventilator dependence, intensive care stays, malnutrition, prolonged parenteral nutritional support, hypoalbuminemia, lymphopenia, immunodeficiency, electrolyte abnormalities, nosocomial infections, and death.^6–8^ Despite severe morbidities and mortality, little is known about the causes of postoperative chylothorax. The etiologies of postoperative chylothoraces have been proposed to be secondary to iatrogenic injury of the thoracic duct, venous or lymphatic congestion, central vein thrombosis, or diffuse injury of mediastinal lymphatic tissue.^9^ There is no recognized standard of care for postoperative chylothorax. Current treatments include conservative management with medium chain triglyceride-enriched diet or total parenteral nutrition in combination with adequate drainage of pleural fluid, octreotide therapy, or surgical intervention in the form of thoracic duct ligation, pleuroperitoneal shunt, or pleurodesis.^10^

As primary chylothorax is observed in patients with lymphatic anomalies,^11^ we hypothesized that patients with congenital cardiac structural defects also have intrinsic lymphatic endothelial cell (LEC) defects. In fact, genes essential for proper cardiac development are also necessary for lymphatic development. In mice, loss of the transcription factor, *Prox1*, a gene necessary for specification and maintenance of LECs,^12^ resulted in muscular septal defects and hypoplastic ventricles.^13^ Similarly, knockout of *Podoplanin*, which is necessary to segregate the blood and lymphatic vasculature,^14–16^ resulted in myocardial defects and cardiac anomalies, as well as chylothorax.^16,17^ These results suggest that there may be subclinical intrinsic LEC defects that contribute to the development of postoperative chylothorax.

We isolated and characterized LECs from postoperative chylothorax fluids secondary to cardiac surgeries, which we termed pcLECs. Expression studies demonstrated that pcLECs misexpressed genes of LECs and endothelial progenitors, suggesting that pcLECs are improperly differentiated. When xenografted in mice, pcLECs formed dilated lymphatic channels, distinctly different from lymphatics developed by control human dermal LECs (HdLECs). Together these data suggest that there are inherent defects in the LECS of congenital cardiac patients that contribute to the postoperative chylothorax.

## Materials and methods

### LEC isolation

Chylous fluid samples were collected from pediatric patients who underwent cardiac surgery to correct structural congenital cardiac deformities (Table 1) with postoperative chylothorax fluid defined as: ≥80% lymphocyte count, and/or chylomicron positivity, and chyothorax fluid triglyerides (TG) > 50% serum TG levels (Columbia University IRB AAAQ6902). Patients with chromosomal anomalies were excluded.

Cells were depleted of CD133+ cells by magnetic bead isolation (Miltenyi) as described.^18^ Nonadherent immune cells were removed after seeding and adherent CD133-negative cells were expanded and characterized by quantitative RT-PCR (qRT-PCR) and fluorescent activated cell sorting (FACS). HdLECs, isolated from neonatal dermis using CD31+ bead selection and live cell sorting for PODOPLANIN, served as normal controls.^19^ pcLECs and HdLECs were maintained on fibronectin-coated plates in EGM-2 media (Lonza) supplemented with 18% FBS or EGM-MV2 media (Lonza), respectively.

### Fluorescence-activated cell sorting

Surface protein expression of pcLECs was determined using antibodies against VECADHERIN, CD31, VEGFR2, PODOPLANIN, VEGFR3, CD90, CD146, and CD45 (Supplemental Digital Table 1, http://links.lww.com/JV9/A9), FACSCalibur flow cytometer with CellQuestPro acquisition software (BD Biosciences, Franklin Lakes, New Jersey), and analyzed with FlowJo software (BD Biosciences, V10.7.1). The peak of expression (decades shift) relative to IgG control antibody was compared to control HdLECs to determine whether there was unchanged, increased, decreased, or absence of cell surface expression.

### Quantitative RT-PCR

Total RNA was isolated from passage 3 to 4 pcLECs or passage 3 to 6 HdLECs with RNeasy Mini-Kit (Qiagen, Germantown, Maryland) and reverse transcribed using First Strand Synthesis Kit (Invitrogen, Waltham, Massachusetts). PCR with Sybr Green Master Mix (ABI, Beverly, Massachusetts) and gene specific primers for *TIE2, PROX1, PODOPLANIN, LYVE1*, and *VEGFC* (Supplemental Digital Table 2, http://links.lww.com/JV9/A9) were done in triplicate on a CFX96 PCR Cycler (Biorad, Hercules, California). Vectors containing amplified region served as control and values were normalized to *β-actin*.

### Murine xenograft model

All animal work was done with an approved protocol from Columbia University’s IACUC (AAAQ8400). The xenograft mouse model was done as previously described.^18,20,21^ Briefly, 1.5 × 10^6^ cells/200 μL Matrige (BD Bioscience, Phenol-Red free, 356237) was injected subcutaneously into the flanks of athymic mice (n = 4; Taconic NcR nude mice, 6–8 weeks of age). Implants were harvested 5 weeks after implantation, formalin-fixed and paraffin-embedded.

### Immunofluorescent staining

Five-micron sections were de-paraffinized, rehydrated, and blocked with donkey serum as previously described.^21^ For cell immunofluorescent (IF) staining, pcLECs and HdLECs were seeded onto a fibronectin-coated *Millicell EZ Slides* (Millipore-Sigma, St Louis, Missouri). After 24 hours, 4% PFA fixed cells were permeabilized, blocked and incubated overnight with primary antibodies at 4°C (Supplemental Digital Table 1, http://links.lww.com/JV9/A9).^21^ Primary antibodies were detected with Alexa Fluor-conjugated anti-donkey-secondary antibodies (Invitrogen) at 1:300 for PODOPLANIN and at 1:1000 for all other antigens. Images were captured with either an Axiocam MRC system (Zeiss, White Plains, New York) or the Olympus IX83 microscope and prepared with Adobe Photoshop.

### Statistics

Significance was determined by Student t test comparing each experimental group relative to vehicle with a *P* value ≤.05 considered significant.

## Results

### Patient demographics

pcLECs were isolated from 10 patients who underwent open heart surgery to repair congenital heart defects and developed postoperative secondary chylothorax (Table 1). In addition, pulmonary tissues were obtained at autopsy from an 11th patient. Cardiac diagnoses included: hypoplastic left heart syndrome (HLHS, n = 5), Shone’s syndrome (n = 1), complete atrioventricular canal defect (n = 1), total anomalous pulmonary venous return (n = 1), Ebstein’s anomaly (n = 1), Taussig-Bing anomaly (n = 1), and Tetralogy of Fallot (n = 1). The median age of surgical repair was 0.3 months (range, 2 d to 5.8 y old). None of the patients were diagnosed with a primary lymphatic anomaly. Four patients were placed on extra-corporeal membrane oxygenation as part of their care, and 1 of 11 patients received a thoracic duct ligation. Two patients died, whereas all others were discharged from the hospital.

### pcLECs misexpressed and mislocalized endothelial proteins

**Figure 1.**
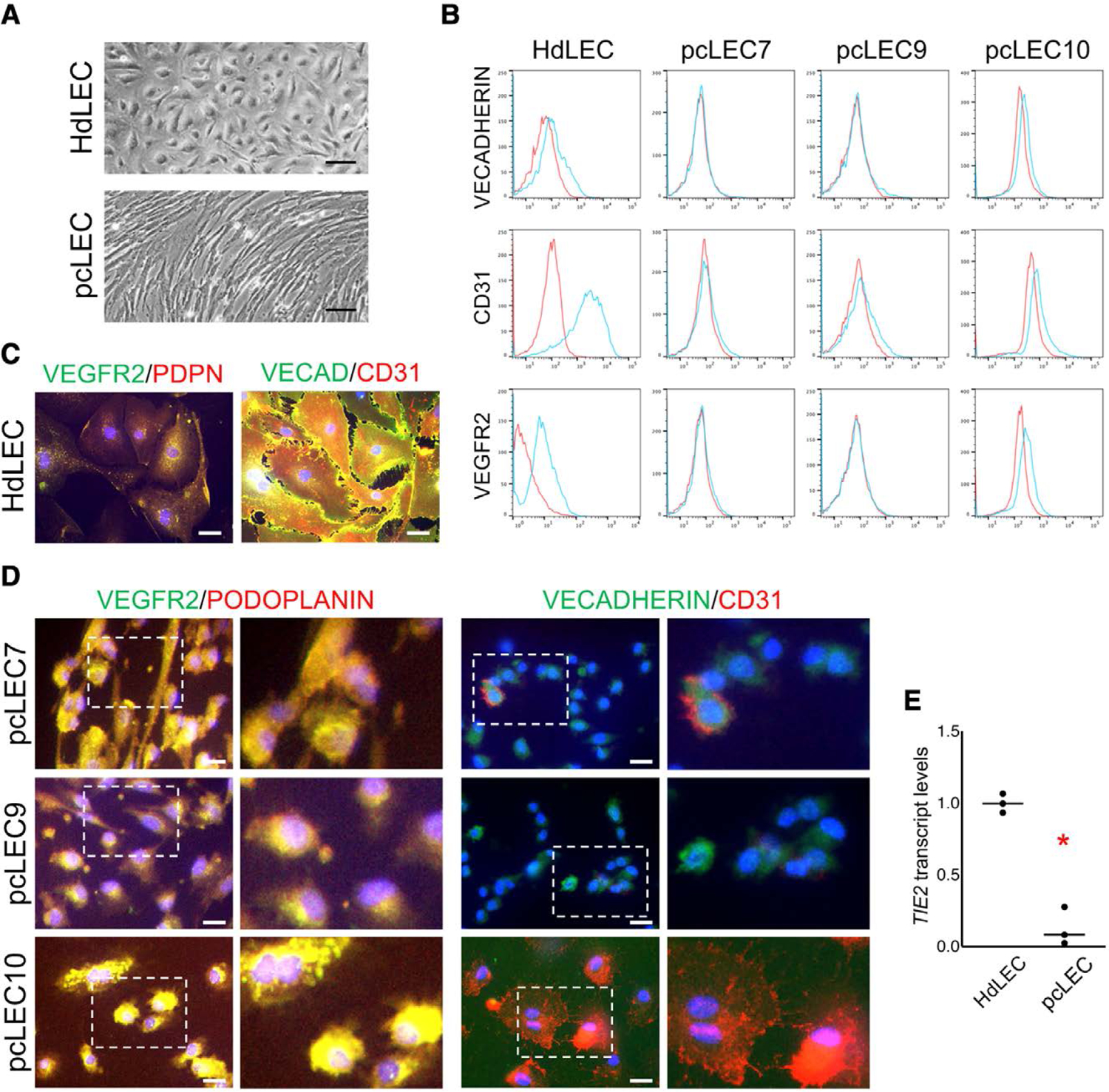
pcLECs expressed endothelial genes. A, Representative HdLECs and pcLECs in culture. B, VECADHERIN, CD31, and VEGFR2 FACS of HdLECs and 3 pcLECs. Blue: specific antibodies, red: IgG control. C, HdLECs and (D) pcLECs immunostained for VEGFR2, PODOPLANIN (PDPN), VECADHERIN (VECAD), and CD31. Scale bars: 50 μm. D, Boxed areas are enlarged to the right. E, *TIE2* qRT-PCR of HdLECs and pcLECs normalized to *β-actin*. Each data point represents a unique cell population (n = 3). *P < .005. Abbreviations: HdLECs, human dermal lymphatic endothelial cells; pcLECs, postsurgical chylothorax lymphatic endothelial cells; qRT-PCR, quantitative RT-PCR.

Ten pcLEC populations were isolated and characterized for the expression of endothelial, lymphatic endothelial, and endothelial progenitor proteins and transcripts. pcLECs did not express CD45, confirming that they were not immune cells (Supplemental Digital Figure 1, http://links.lww.com/JV9/A9). Unlike control HdLECs which form tight cell–cell junctions with neighboring LECs, pcLECs were poorly associated with an altered elongated cell morphology (Figure 1A). FACS did not detect cell surface expression of CD31 (4/10), VECADHERIN (7/10), and VEGFR2 (2/10) in the majority of pcLEC populations (Figure 1B; Table 2). pcLEC populations also had decreased CD31 (6/10) and VEGFR2 (8/10) cell surface expression relative to HdLECs (Table 2). IF staining revealed that 3 pcLECs expressed comparable if not higher levels of VEGFR2 in their cytosol relative to HdLECs (Figure 1C, D). VECADHERIN and CD31 expression, which was reduced, was also observed in the cytosol of pcLECs (Figure 1C, D). pcLECs did express *TIE2* transcripts, but they were significantly decreased relative to HdLECs (Figure 1E). Taken together, pcLECs expressed endothelial specific genes/proteins, but they were often reduced in expression or mislocalized relative to wild-type LECs.

### pcLECs expressed lymphatic endothelial genes

**Figure 2.**
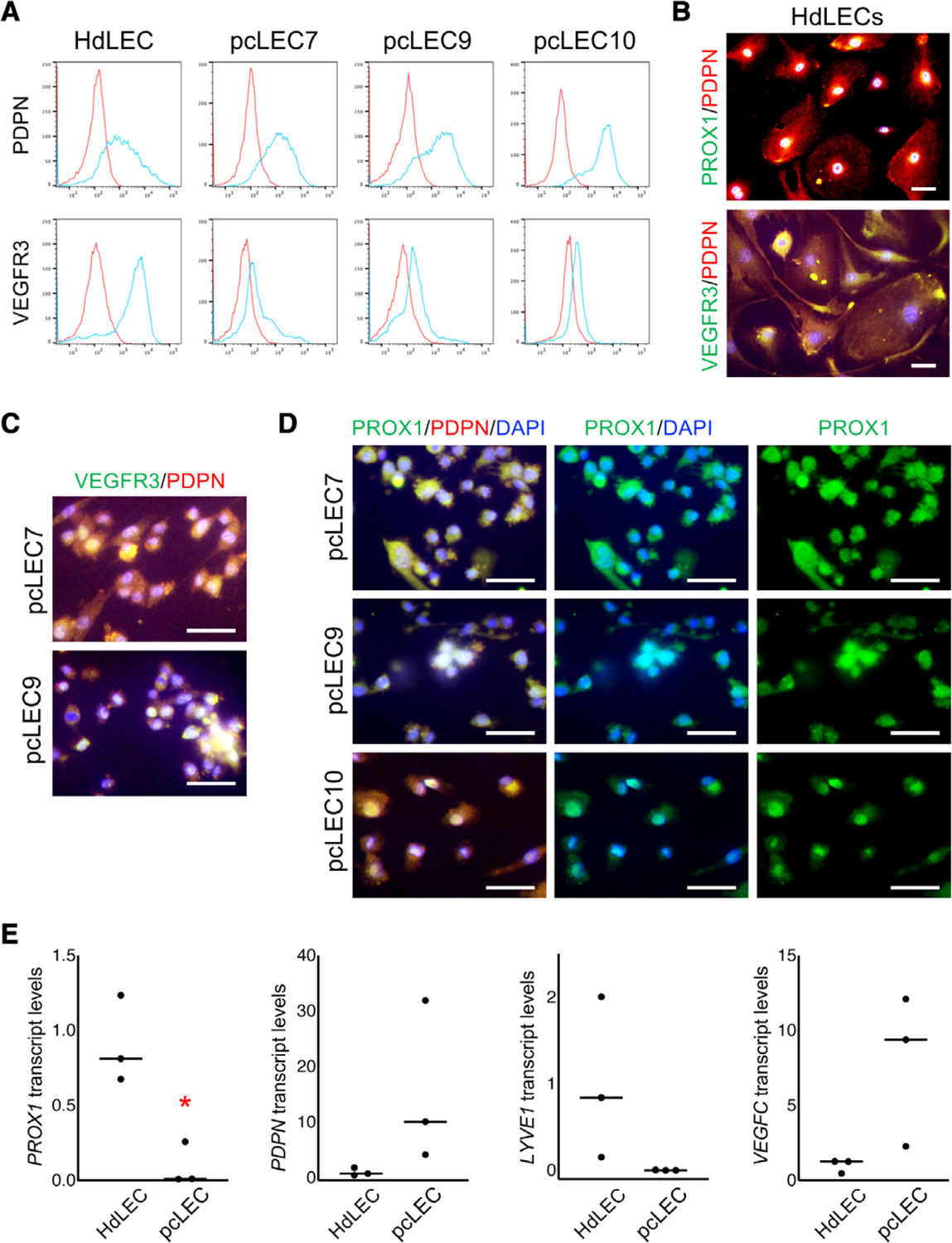
pcLECs expressed LEC genes. A, PODOPLANIN and VEGFR3 FACS of HdLECs and 3 pcLECs. Blue: specific antibodies, red: IgG control. C, HdLECs immunostained for PODOPLANIN (PDPN), PROX1, and VEGFR3. pcLECs immunostained for (C) PDPN and VEGFR3 or (D) PDPN and PROX1. B–D, Scale bars: 50 μm. E, *PROX1*, *PODOPLANIN*, *LYVE1*, and *VEGFC* qRT-PCR of HdLECs and pcLECs normalized to *β-actin*. Each data point represents a unique cell population (n = 3). *P < .005. Abbreviations: HdLECs, human dermal lymphatic endothelial cells; pcLECs, postsurgical chylothorax lymphatic endothelial cells.

pcLECs were next evaluated for the expression of lymphatic endothelial genes and proteins (Figure 2). HdLECs expressed PODOPLANIN and VEGFR3 (Figures 1C and 2A, B), and PROX1 in the cell nucleus (Figure 2B). A majority of pcLECs (9/10) expressed PODOPLANIN (Figures 1D and 2A, C, D) similar to HdLECs at both protein (Table 2) and transcript levels (Figure 2E). Unlike PODOPLANIN, cell surface expression of VEGFR3 was decreased (9/10) or absent (1/10) in all pcLEC populations (Figure 2A; Table 2). Similar to VEGFR2 (Figure 1D), pcLECs without surface VEGFR3 expression had cytosolic VEGFR3 (Figure 2C). *PROX1*, a transcription factor necessary to induce and maintain LEC identity, was expressed in pcLECs (Figure 2D), though its expression relative to HdLECs was significantly lower (Figure 2E). IF revealed that, unlike HdLECs where PROX1 was mostly localized to the nucleus (Figure 2B), PROX1 expression was observed highest in the cytosol of pcLECs (Figure 2D). Finally, *LYVE1* transcripts were not detected or low in pcLECs relative to HdLECs. Although it did not achieve significance, *VEGFC* transcripts trended higher than HdLECs (Figure 2E, *P* = .08). These data demonstrate that pcLECs express LEC genes and proteins, but they are often expressed at lower levels and mislocalized to the cytosol.

### pcLECs express progenitor endothelial genes

**Figure 3.**
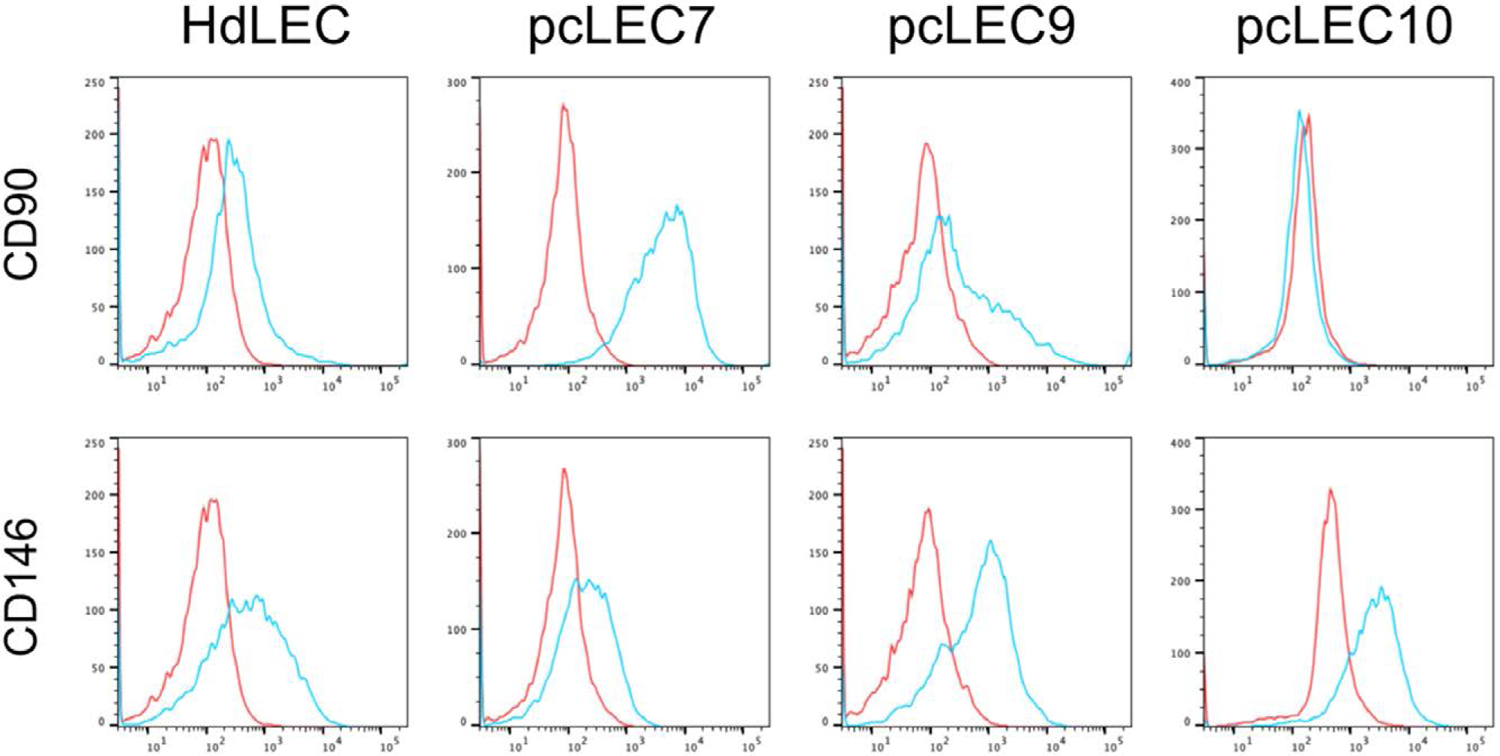
pcLECs expressed progenitor EC genes. CD90 and CD146 FACS of HdLECs and 3 pcLECs. Blue: specific antibodies, red: IgG control. Abbreviations: HdLECs, human dermal lymphatic endothelial cells; pcLECs, postsurgical chylothorax lymphatic endothelial cells.

We have previously shown that the LECs from congenital lymphatic anomalies express endothelial progenitor proteins, CD146 and CD90.^18^ A majority of pcLECs (8/10) had increased CD90 (Table 2), whereas 2 of 10 had decreased CD90 (Figure 3; Table 2). Half of the pcLECs expressed CD146 similar to HdLECs, whereas the other half had reduced to absent expression (Figure 3; Table 2). In summary, pcLECs expressed endothelial progenitor cell maker, CD90, with variable CD146 expression observed.

### pcLECs developed dilated and discontinuous lymphatic channels in murine xenografts

**Figure 4.**
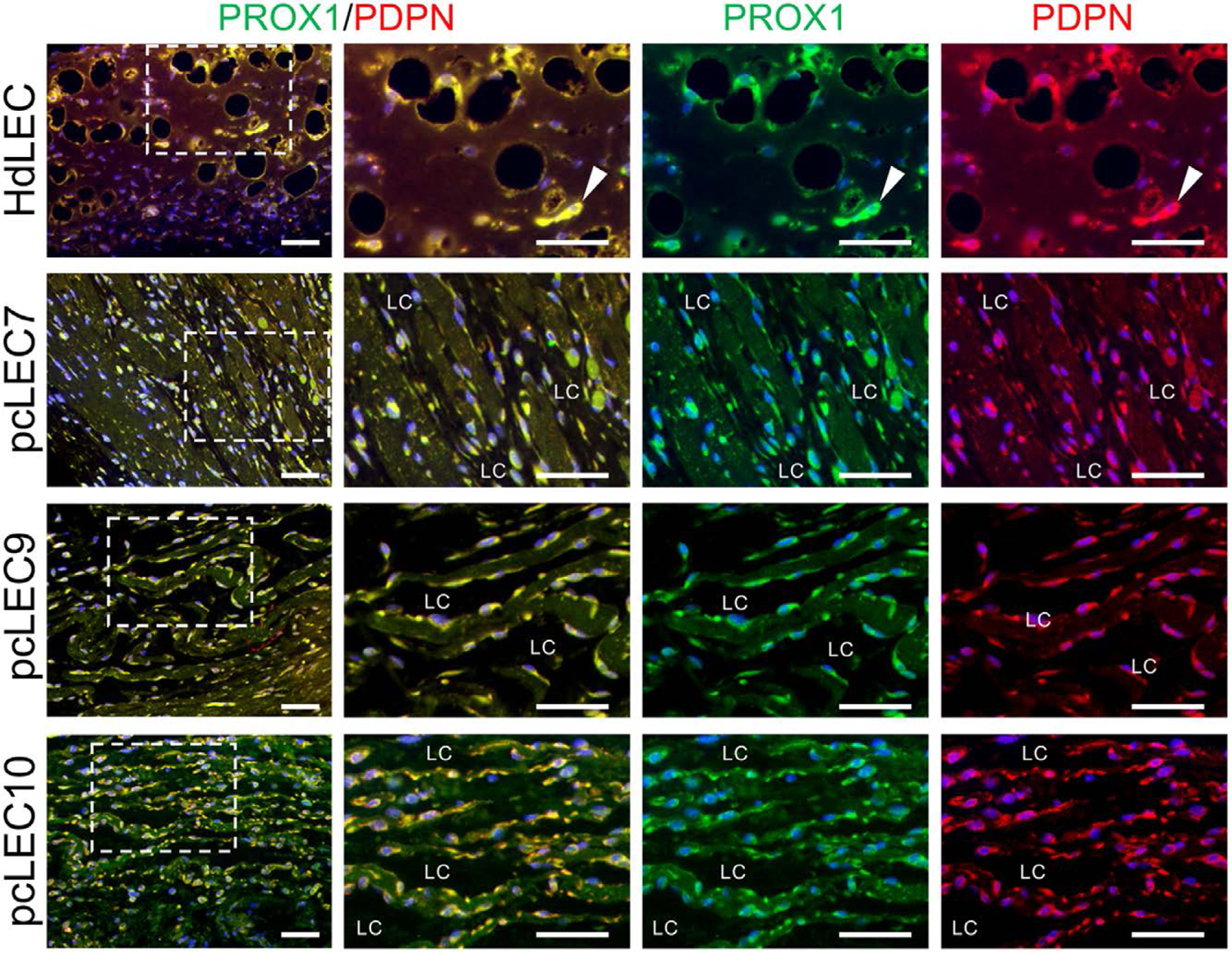
pcLECs form abnormal lymphatic channels in a xenograft mouse model. Five-week HdLECs and pcLEC implants were immunostained for PROX1 and PODOPLANIN. Boxed areas in images in the first column are enlarged to the right. White arrowheads mark lymphatic vessels. Scale bar, 50 μm. Abbreviations: HdLECs, human dermal lymphatic endothelial cells; LC, lymphatic channel; pcLECs, postsurgical chylothorax lymphatic endothelial cells.

**Figure 5.**
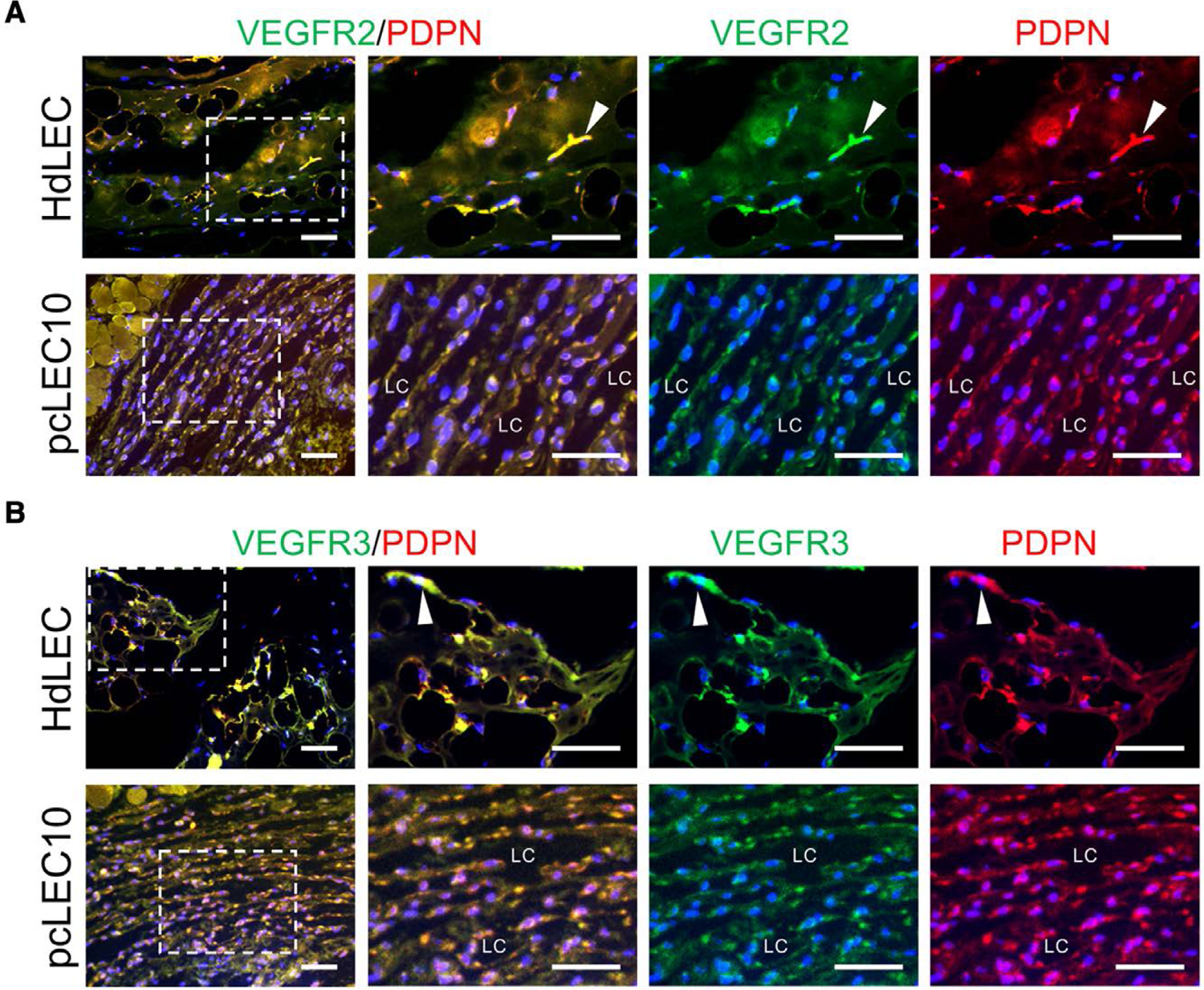
pcLEC-derived lymphatic channels expressed VEGFR2 and VEGFR3. Control HdLECs and pcLECs implants stained for (A) VEGFR2 and PODOPLANIN (PDPN), or (B) VEGFR3 and PDPN. Boxed areas in images in the first column are enlarged to the right. White arrowheads mark lymphatic vessels. Scale bar, 50 μm. Abbreviations: HdLECs, human dermal lymphatic endothelial cells; LC, lymphatic channel; pcLECs, postsurgical chylothorax lymphatic endothelial cells.

To assess lymphatic vessel formation in vivo, 3 pcLEC populations or HdLECs were resuspended in Matrigel and injected subcutaneously into the flanks of immunocompromised mice. The lymphatic vessel phenotype was assessed after 5 weeks by immunostaining for PROX1, PODOPLANIN, VEGFR3, and VEGFR2. Double staining with PROX1 and PODOPLANIN confirmed that the channels are lined by LECs (Figure 4). When compared to control HdLECs, pcLECs formed numerous large and dilated vascular channels lined by LECs with poor cell–cell junctions. LECs lining the lymphatic channels also expressed VEGFR2 and VEGFR3 (Figure 5), confirming the channels are lined by LECS that expressed PROX1, PODPODOPLANIN, VEGFR2, and VEGFR3.

### Pulmonary lymphatic vessel defects in a congenital heart patient with severe postsurgical chylothorax

**Figure 6.**
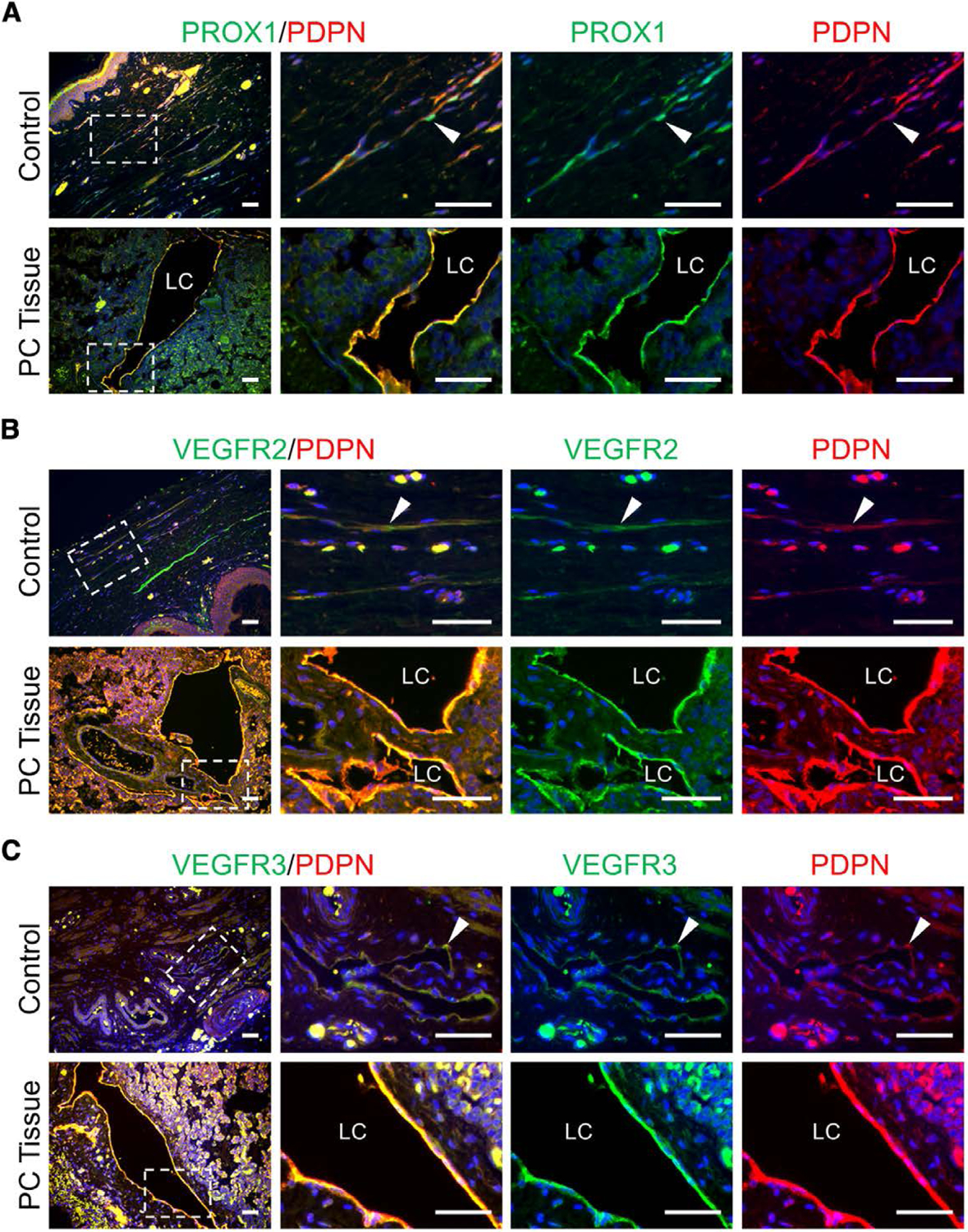
Abnormal lymphatic vasculature in patient with secondary chylothorax. Pulmonary tissues obtained from a patient who developed secondary chylothorax after cardiac surgery (PC tissue) was compared to neonatal dermis (Control). Tissue sections were immunostained for (A) PROX1 and PODOPLANIN (PDPN), (B) VEGFR2 and PDPN, or (C) VEGFR3 and PDPN. Boxed areas in images in the first column are enlarged to the right. White arrowheads mark lymphatic vessels. Scale bar, 50 μm. Abbreviation: LC, lymphatic channel.

The pcLEC phenotype in vitro and in vivo suggested that patients with congenital structural cardiac defects had underlying, subclinical lymphatic anomalies which were unmasked due to fluid overload or fluid shifts secondary to surgery. The pulmonary tissues of a patient, born with HLHS, aortic arch hypoplasia, and valvular anomalies and developed postoperative chylothorax after aortic arch, and supravalvar aortic stenosis repair was stained for PROX1, PODOPLANIN, VEGFR2, and VEGFR3. When compared to control neonatal dermis, the lymphatic endothelium, which expressed PROX1, PODOPLANIN, VEGFR3, and VEGFR2, was dilated and disorganized (Figure 6).

## Discussion

Our results demonstrate that LECs can be isolated from postoperative chylothorax fluids from cardiac defect patients. These pcLECs expressed endothelial (CD31, VECADHERIN, and VEGFR2), lymphatic endothelial (PROX1, PODOPLANIN, and VEGFR3), and endothelial progenitor cell markers (CD90 and CD146), though these proteins were often expressed at lower levels and mislocalized relative to LECs from healthy donors. Expression of CD31, VEGFR2, and VEGFR3 was decreased at the cell surface of all pcLECs. However, the expression of some proteins between pcLECs from different patients was heterogenous. VECADHERIN expression at the surface was seen in a small portion of pcLECs, whereas expression of the progenitor cell markers, CD90 and CD146, was variable. This heterogeneity in the expression patterns between patient pcLECs may be due to the differences in the genetic or epigenetic defects contributing to this pathology. In a xenograft model, pcLECs formed disorganized vascular channels lined by cells that expressed multiple LEC proteins. The mislocalization of adhesion proteins CD31 and VECADHERIN may explain why these pcLECs could be isolated from the chylous effusions and formed discontinuous lymphatic channels when xenografted. We also observed reduced expression and mislocalization of PROX1, a gene necessary to maintain LEC identity and function, suggesting pcLECs fail to properly differentiate. Together these data suggest that pcLECs have inherent defects that contribute to the development of abnormal dilated lymphatic channels in secondary chylothorax in patients with congenital heart defects. Consistent with this hypothesis, we observe dilated ectatic lymphatic vessels in lung tissue from a patient with HLHS and aortic arch and valvular anomalies that developed secondary chylothorax.

Our findings suggest that some congenital heart defect patients have inherent, subclinical lymphatic anomalies that are unmasked during the postoperative period after cardiac surgery. Supporting this idea, there is an association between lymphatic anomalies and cardiac defects that occur in patients with chromosomal anomalies such Down syndrome and Turner syndrome.^22,23^ Similarly, patients with Noonan syndrome, which is associated with germline mutations in genes leading to activation of the RAS/RAF/MAPK pathway, often have both heart defects and lymphatic anomalies.^24^ Burger et al^25^ performed a review of Pubmed and Mammalian Phenotype Browser for genes that function in heart and lymphatic development and identified candidate genes; *ADRENOMEDULLIN*, *COUPTFII*, *CYP51*, *EPHRINB2*, *FOXC2*, *NFATC1*, *NF1*; *PODOPLANIN*, *PROX1*, *PIK3CA*, *TBX1*, *TIE1*, *VEGFA*, *VEGFR3* and *VEZF1*. Murine studies of *Prox1*, *Podoplanin*, and *CouptfII (Nr2f2*) have shown these mice develop both cardiac and lymphatic defects.^13,16,17,26–28^ Cardiac-specific deletion of *Prox1* knockout mice develop structural cardiac anomalies including deceased heart size, hypoplastic ventricular, and disorganized interventricular septum,^13^ while global deletion of *Prox1* leads to severe lymphatic defects and embryonic lethality.^26^ Mice deficient for *Podoplanin* develop smaller hearts with defects in the aorta, myocardium, ventricular septum, atrioventricular cushions, and coronary arteries.^17^ Analysis of the lymphatic phenotype in *Podoplanin-*deficient mice revealed a failure to separate the blood and lymphatic vasculature with a portion of animals developing postnatal chylothorax.^16^ In mice, *CouptfII* is necessary for the development of LECs progenitors in the venous system and its loss leads to mass edema.^27^ Mutations in *CouptfII* (*Nr2f2*) are associated with Shone’s syndrome in humans and a failure of the development of the atria and sinus venosus in mice.^29^ *CouptfII* variants have also been identified in patients with HLHS, atrioventricular septal defects and coarctation of the aorta.^30^ Taken together, the human genetics and murine studies suggest that some congenital cardiac patients have genetic mutations that lead to both heart and lymphatic defects that contribute to the development of postoperative chylothorax.

Recent studies using advanced lymphatic imaging techniques have demonstrated structural and flow anomalies in the central lymphatic system in patients with congenital cardiac anomalies. A retrospective study of lymphangiograms in patients who underwent surgical correction of single ventricle abnormality found that >85% of patients had anatomic lymphatic anomaly.^31^ A study by Savla et al^32^ demonstrated that only a minority (8%) of congenital cardiac patients developed postoperative chylothorax after cardiac surgery as a result of traumatic injury to the thoracic duct (TD). Of the remaining patients, 14 (56%) had abnormal lymphatic flow from the TD into lung parenchyma through abnormal lymphatic networks channels, and 9 (36%) had central lymphatic flow disorder. In central lymphatic flow disorder, patients had abnormal backflow via dermal lymphatic collaterals. That is, 92% of postoperative chylothorax patients had intrinsic anatomic and/or physiologic lymphatic anomalies in the lung parenchyma resulting in abnormal lymph flow and backflow in this patient population.

Expression studies of human lung LECs are limited. Both the lymphatics surrounding the major arteries and intralobular lymphatic vessels express PODOPLANIN.^33^ Similar to HdLECs used in these studies, LECs isolated from lungs from cancer patients express PROX1 in the nucleus, and PODOPLANIN, VEGFR2, CD31, and LYVE1 at the cell surface.^34^ pcLECs had reduced expression or mislocalization of proteins necessary for LEC differentiation and function, suggesting the anatomical and physiologic lymphatic vascular defects observed by imaging may be a result of these LEC defects. Similar to LECs isolated from congenital lymphatic anomalies,^18,35,36^ pcLECs had increased *VEGFC* and VEGFR3 expression,^35,36^ mislocalized endothelial junctional proteins, CD31 and VECADHERIN,^18,37^ and formed dilated lymphatic channels when xenograft in mice.^18,37^ Since pcLECs come from cardiac patients who developed postoperative chylothorax, their similarity to LECs from congenital lymphatic anomalies further supports that there are intrinsic LEC defects in congenital cardiac patients.

We postulate that the intrinsic lymphatic anomalies in congenital cardiac patients are on the milder, subclinical spectrum which is exacerbated by changes in fluid balance or shifts experienced by congenital heart defect patients. Altered lymphatic physiology secondary to prolonged and increased pulmonary blood flow associated with congenital cardiac defects has been reported.^38^ In a lamb model, chronic, prolonged increases in pulmonary blood flow were associated with the development of dilated pulmonary lymphatics and reduced lymphatic flow.^38^ It has also been demonstrated that the Fontan procedure, used to correct hypoplastic left heart syndrome, is associated with increased stress on the lymphatic system due to the elevation in systemic venous pressure.^39^ Similarly, Itkin et al^40^ proposed that increased lymphatic flow coupled with elevated central venous pressures close to serous surfaces results in pleural chylothorax. In summary, postoperative chylothorax could be a direct result of increased pressure into the lymphatics from chronically elevated central venous blood flow putting stress on an inherently, anatomically abnormal lymphatic system.

## Conclusion

Lymphatic anomalies should be considered a component of congenital cardiac disease, whether it is clinically apparent or not. Even when it is subclinical, the lymphatic system can be affected from cardiac procedures that correct the congenital cardiac deformities, leading to postoperative chylothorax, a significant morbidity.

## Supplementary Material

Supplemental Material

## Figures and Tables

**Table 1.** Patient Demographics

Patient	Diagnosis	Operation	Age at Operation (mo)	Pre-existing lymphatic anomaly	Outcome
1	HLHS	Fontan	34	No	Discharged from hospital
2	Shone’s syndrome	Arch repair, VSD closure	0.3	No	Discharged from hospital
3	HLHS	Fontan	50	No	Discharged from hospital
4	CAVC	CAVC repair	1	No	Died
5	HLHS	Norwood	0.17	No	Discharged from hospital
6	TAPVR	TAPVR repair	0.07	No	Discharged from hospital
7*	Ebstein’s anomaly	Tricuspid valve repair, Bidirectional Glenn	70	No	Discharged from hospital
8	Taussig-Bing anomaly	Arterial switch, RVOT patch plasty	0.23	No	Discharged from hospital
9*	HLHS	Bidirectional Glenn	4	No	Discharged from hospital
10*	ToF	ToF repair	0.27	No	Discharged from hospital
11†	Hypoplastic left heart; Coarctation of aorta	Arch repair with supravalvular repair Norwood	0.27	No	Died

* pcLECs used for qRT-PCR, IF, and xenograft model.

† Tissue only; no cells.

Abbreviations: CACD, complete atrioventricular canal defect; HLHS, hypoplastic left heart syndrome; pcLECs, postsurgical chylothorax lymphatic endothelial cells; TAPVR, total anomalous pulmonary venous return; ToF, Tetrology of Falllot

**Table 2.** Summary of pcLEC FACS Results Relative to HdLEC

Antigen	No Change	Increased	Decreased	Absent
VECADHERIN	3/10			7/10
CD31			6/10	4/10
VEGFR2			2/10	8/10
PODOPLANIN	5/10	4/10	1/10	
VEGFR3			9/10	1/10
CD90		8/10	2/10	
CD146	5/10		2/10	3/10

Abbreviations: FACS, fluorescent-activated cell sorting; HdLECs, human dermal lymphatic endothelial cells; pcLECs, postsurgical chylothorax lymphatic endothelial cells.
