# Supplementary material for: Lymphatic Endothelial Cell Defects in Congenital Cardiac Patients With Postoperative Chylothorax
Source: J Vasc Anom (Phila). Author manuscript; Available in PMC 2021 Sep 28. (PMC8478352; doi:10.1097/jova.0000000000000016)
Supplement: Supplemental Material [file NIHMS1739977-supplement-Supplemental_Material.pdf]

Shakoor SDC Figure 1

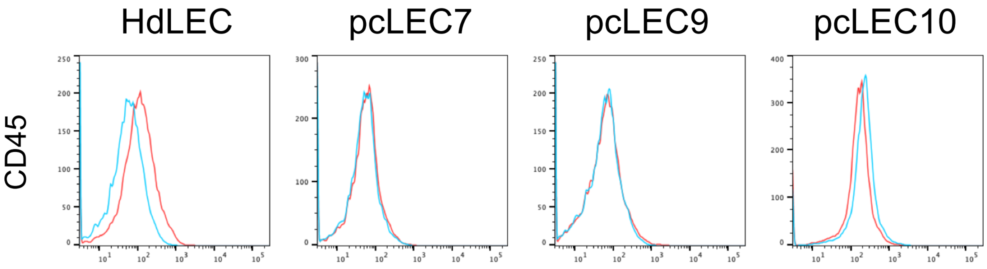

SDC Figure 1. pcLECs are not immune cells. CD45 FACS of HDLECs and 3 pcLECs. blue: specific antibodies, red: IgG control

SDC, Table 1. Summary of RT-PCR gene specific primers.

Gene	Forward Primer	Reverse Primer
β-actin	5' CGAGGCCCAGAGCAAGAGAG 3'	5' CTCGTAGATGGGCACAGTGTG 3'
TIE2	5’GAAGATGCAGTGATTTACAAAAATG3’	5’AGCCTGTGGCACAGGAACAC 3’
PROX1	5' ACGTAAAGTTCAACAGATGCATTAC 3'	5' CCAGCTTGCAGATGACCTTG 3'
PODOPLANIN	5' CCCAGGAGAGCAACAACTCAAC 3'	5' CTCGATGCGAATGCCTGTTAC 3'
LYVE1	5' TAGCTTTGAAACTTGCAGCTATG 3'	5' TCAACAAATGGTTCAGTTTCTGTAG 3'
VEGFC	5' GCCACGGGAGGTGTGTATAGA TG 3'	5' TGTTCGCTGCCTGACACTGTG 3'

SDC Table 2. Summary of Antibodies

Antigen	FACS	Immunofluorescence
VECADHERIN	RnD Systems (FAB9381P)	RnD Systems (AF938)
CD31	Ancell (180-040)	DAKO (M082)
CD45	RnD Systems (FAB1430P)	
CD90	BD Biosciences (555595)	
CD146	ThermoFisher (11-1469-42)	
PODOPLANIN	EBioscience (17-9381-42)	RnD Systems (AF3670)
PROX1		RnD Systems (AF2727)
VEGFR2	RnD Systems (FAB357P)	RnD Systems (AF357)
VEGFR3	RnD Systems (FAB3492P)	RnD Systems (AF349)
